# A fast linear predictive adaptive model of packed bed coupled with UASB reactor treating onion waste to produce biofuel

**DOI:** 10.1186/s12934-016-0563-y

**Published:** 2016-10-03

**Authors:** Harvey Milquez-Sanabria, Luis Blanco-Cocom, Liliana Alzate-Gaviria

**Affiliations:** 1 Renewable Energy Unit, Yucatan Center for Scientific Research (CICY), Calle 40 No. 130, Colonia Chuburná de Hidalgo, 97200 Mérida, Yucatán Mexico; 2 Mathematics Research Center, A.C., Jalisco S/N, Colonia Valenciana, 36023 Guanajuato, Gto Mexico

**Keywords:** Predictive adaptive model, Onion waste, Coupled system, Methane, Anaerobic digestion

## Abstract

**Background:**

Agro-industrial wastes are an energy source for different industries. However, its application has not reached small industries. Previous and current research activities performed on the acidogenic phase of two-phase anaerobic digestion processes deal particularly with process optimization of the acid-phase reactors operating with a wide variety of substrates, both soluble and complex in nature. Mathematical models for anaerobic digestion have been developed to understand and improve the efficient operation of the process. At present, lineal models with the advantages of requiring less data, predicting future behavior and updating when a new set of data becomes available have been developed. The aim of this research was to contribute to the reduction of organic solid waste, generate biogas and develop a simple but accurate mathematical model to predict the behavior of the UASB reactor.

**Results:**

The system was maintained separate for 14 days during which hydrolytic and acetogenic bacteria broke down onion waste, produced and accumulated volatile fatty acids. On this day, two reactors were coupled and the system continued for 16 days more. The biogas and methane yields and volatile solid reduction were 0.6 ± 0.05 m^3^ (kg VS_removed_)^−1^, 0.43 ± 0.06 m^3^ (kg VS_removed_)^−1^ and 83.5 ± 9.8 %, respectively. The model application showed a good prediction of all process parameters defined; maximum error between experimental and predicted value was 1.84 % for alkalinity profile.

**Conclusions:**

A linear predictive adaptive model for anaerobic digestion of onion waste in a two-stage process was determined under batch-fed condition. Organic load rate (OLR) was maintained constant for the entire operation, modifying effluent hydrolysis reactor feed to UASB reactor. This condition avoids intoxication of UASB reactor and also limits external buffer addition.

## Background

According to the Food and Agriculture Organization of the United Nations (FAO), Japan, Republic of Korea and China are the highest producers of onions in the world with over 1500 million tons per year, equivalent to 65 % of the world production [1]. In 2014, Mexico had a production over 1.3 million tons per year; 85 % of the production was used in domestic market [2]. Likewise, for thousands of years, agriculture was a natural process that did not harm the land it was done on. In fact, farmers were able to pass down their land for many generations and it would still be as fertile as ever. However, modern agricultural practices and irrigation systems have started the process of agricultural pollution. This process causes the degradation of the eco-system, land and environment due to the by-products of agriculture [3–5]. The wastes are roots, discarded bulbs, dried leaves, peels, skins and stems, usually generated during agro-industrial packaging. Actually, onion wastes are disposed of mainly by means of animal food, landfills, dumps or incinerators. Several studies have reported alternatives for this waste: antioxidant for food industry [6, 7], composting [8, 9], pharmacological ingredients [10], antimicrobial extraction compounds [10, 11], production of vinegar [12–14], organic fertilizer [15] and energy production [16, 17].

Alternative energy has become important for world energy stability, environmental protection and developing countries [18]. Agro-industrial solid wastes are still a potential energy resource if they can be properly and biologically converted to methane [18, 19]. Actually, large quantities of agro-industrial wastes are destined for landfills, thereby reducing their usefulness [20, 21].

Anaerobic digestion consists of three steps: hydrolysis, acidogenic and methanogenic [22]. In the two first phases complex organic components are hydrolyzed and fermented into intermediate volatile fatty acids (VFA). In the final phase those VFA are reduced to methane and carbon dioxide [23]. A major limitation of anaerobic digestion in a single-phase is a large production of VFA and decreased pH of the solution, causing inhibition of the methanogenic bacterial community [24]. The formation of organic acids, H_2_ and methane production can be separated into separate bioreactors in series in which the first produces organic acids, H_2_ and CO_2_, while the second produces CH_4_ and CO_2_ [25].

The total retention time in the two-phase system is shorter than in the single phase. Furthermore, the gas conversion efficiency and methane concentration in the biogas are higher [26]. And finally, this process can be suitable for better process control. To increase the conversion of organic waste, both cellulose and hemicellulose need to be broken down to monosaccharides. This pretreatment is an important step for lignocellulosic biomass, including chemical, physical and biological processes [27–29].

Some authors have used rumen microorganisms for the degradation of onion waste, but the maximum loading rate was lower than for lignocellulosic substrates. Those authors suggested that high amounts of easily degradable sugars result in a relatively low acetate concentration which benefits butyric acid and high FA [30]. Other authors evaluated the performance of a mixed biofilm anaerobic digester (AMBR) for treating the mixture of onion juice and aerobic sludge under different mixing ratios and organic load rates. They found that under batch conditions the total biogas yield was 0.62 L gVS^−1^ with a concentration of methane over 60 % [16]. Likewise, they determined the digestibility of onion residuals using a two-phase anaerobic phased solid digester: one anaerobic mixed biofilm reactor for hydrolysis and a packed bed reactor for methanogenic phase. The study showed that the process is possible with the application of external chemicals for maintaining the alkalinity and pH. Biogas yield of the entire system was 0.69 L gVS^−1^ with a concentration of methane between 60 and 70 % [17].

On the other hand, there are advanced models which are based on complex knowledge in waste character and kinetics. These models require extensive analytical solving of sequential reactions and intermediate products where environmental factors are an effect; therefore, fast response producing models are needed for ideal control strategies. In fact, one of the most commonly used empirical models has been the anaerobic digestion model number 1 (ADM1) [31]. However, these approaches may involve many constant parameters with values that are specific and need to be sought from experiments or through assumptions. In addition, these constants are assumed to be the same forever; this may not be true in the presence of ever changing internal and external conditions [32]. The main difference of the proposed model and conventional statistical or empirical base model is that the coefficients are updated at every time step, providing adaptive ability in the presence of changing conditions over real time.

The objectives of this study were to determine a linear predictive adaptive model (LPAM) for anaerobic digestion in two stages of onion waste and analyses performance in terms of volatile fatty acids (VFA), alkalinity, ammonia and total nitrogen, biogas yield and methane concentration.

## Methods

### Characterization of onion waste

Onion waste was collected from wholesale distributors located in Mérida, Yucatán, México, and delivered to the renewable energy unit at Yucatan center for scientific research (CICY). The onion residues were analyzed for moisture content (MC), total solids (TS) and volatile solids (VS) using standard methods [33] before and after treatment. The sample was analyzed for carbon (C) and nitrogen (N) in the Fish Nutrition Lab of CINVESTAV—Mérida, Yucatán, and fibers (lignine, cellulose and hemicellulose), in the department of Wood, Cellulose and Paper in Guadalajara, Jalisco, according to the TAPPI standard test.

### Chemical analyses

The biogas phase was monitored daily for CH_4_ and volume; likewise, liquid phase was monitored daily for pH, VFA, and ammonia nitrogen and COD (Chemical Oxygen Demand). Biogas composition was measured using a gas chromatography Clarus 500-Perkin Elmer with the thermal conductivity detector (TCD), a Molesieve column (30 m long, 0.53 mm internal diameter and 0.25 μm film thicknesses), nitrogen as the carrier gas and temperatures of 75, 30 and 200 °C for the injector, oven and detector, respectively [34]. The pH was determined by a HQ-40d multi pH-meter. The VFA was determined by titration with H_2_SO_4_ [35]. COD, total nitrogen (N_T_), and ammonia nitrogen (N-NH_3_) were determined via colorimetric methods (HACH Company DR-890).

### Reactor characteristics

The hydrolysis reactor (HPR) was made with acrylic (diameter: 14.5 cm, height: 26.4 cm, total volume: 4.35 L, useful volume: 2.2 L); one screen was fitted at the bottom side of the reactor to collect the leachate. The reactor was filled with a mix of onion waste and PVC plastic rings (1 inch diameter and 1 cm wide) to increase the porosity of the packed bed and facilitate the percolation of the leachate. At the top side of the reactor a sample port for the biogas determination was placed and its volume was registered daily through a gasometer. An Iwaky EZBD1 peristaltic pump was used for maintaining the re-circulation. This reactor was maintained at laboratory temperature (25 °C ± 5).

The methanogenic reactor (UASB) was made with PVC (diameter: 10.2 cm, height: 68 cm, total volume: 5.5 L, useful volume: 5 L) and was inoculated with a mixture of non-anaerobic anaerobic seeds: 300 gL^−1^ cattle manure, 150 gL^−1^ of pig manure, 1.5 gL^−1^ of sodium carbonate and 1 L tap water, according to Blanco-Cocom [36], and was maintained at 35 °C by a regular water bath (Lauda Alpha RA 8). Biogas produced passed to a Ritter MGC-10 milligas counter for volume determination. A peristaltic pump (Iwaky EZBD1) was used for maintaining liquid up flow velocity of 1 ms^−1^.

### Hydrolysis reactor operation

The first hydrolysis reactor was a single-phase reactor (HPR1) and was run in batch mode. This reactor was filled with PVC rings, 460 g of onion waste, diced pieces about 1 cm^2^ and 0.6 L of inoculum. The second hydrolysis reactor (HPR2) was filled with PVC rings, 460 g onion waste, diced pieces about 1 cm^2^, and 0.4 L solution of 1 M H_2_SO_4_ which was recycled for 1 day. Then, the leach was neutralized by adding Na_2_CO_3_ and 0.6 L inoculum. Both reactors were performed in duplicate.

### UASB start-up

The UASB reactor had an initial acclimatization period with an OLR (Organic Loading Rate) of 0.81 ± 0.02 kg VS (m^3^d)^−1^ using 0.2 Ld^−1^ synthetic wastewater (SW) as mentioned in Alzate-Gaviria [37]. After 30 days the OLR was 2.5 ± 0.08 kg VS (m^3^d)^−1^, using 0.4 Ld^−1^ of SW (Methane Yield: 0.32 ± 0.01 m^3^ kg ${\text{COD}}_{\text{removed}}^{ - 1}$) [38, 39].

### Coupled system

OLR was the connection parameter between two reactors, according to Lehtomäki [40] and Yu [26]. The HPR2 was chosen to be coupled to the UASB because its performance obtained the shortest time. OLR was kept constant through measuring COD effluent daily of HPR2, which together with hydraulic retention time of UASB allowed for daily calculation of the volume fed to UASB.

## Model development

A linear predictive adaptive model (LPAM) was used to model the relationship between two variables for fitting a linear equation to observed and predicted data the next set of state. It considers the experimental input values U in time t, the output variable y, the state variables X in time t + 1 and ε due to experimental and numerical errors. The relation between them is given by the following equations:

1 $${\mathbf{X}}\left( {{\text{t}} + 1} \right) = {\mathbf{U}}\left( {\text{t}} \right){\mathbf{A}} + {\varepsilon }$$

2 $${\mathbf{y}}\left( {{\text{t}} + 1} \right) = {\mathbf{U}}\left( {\text{t}} \right){\mathbf{b}} + {\varepsilon }$$

**Fig. 1:**
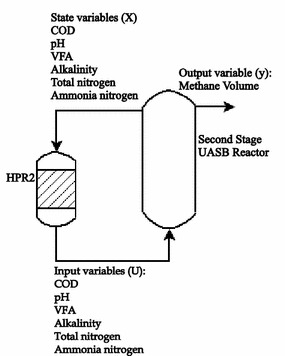
Experimental Setup

The model was built for the coupled HPR2 + UASB reactors. Operating conditions for UASB were input variables from HPR2 (**U**), output methane volume (**y**) and effluent UASB condition state variable (**X**); determination of matrix coefficient **A** and vector **b** are explained in the next lines. The process flow diagram of the two-phase anaerobic system with process variables are shown in Fig. 1.

### Estimation of coefficients via multiple linear regression

Consider Eqs. (1) and (2) where **X** is the matrix defined by the x_i,t_ states (3); **U** is the matrix defined by the u_i,t_ input variables (4) where i is the variable (COD, pH, alkalinity, VFA, nitrogen); t is time; **y** is the methane volume output vector (5) and ε the error.

3 $${\mathbf{X}} = \left[ {\begin{array}{*{20}c} {{\text{x}}_{1,1} } & \cdots & {{\text{x}}_{{{\text{n}},1}} } \\ \vdots & \ddots & \vdots \\ {{\text{x}}_{{1,{\text{t}} - 1}} } & \cdots & {{\text{x}}_{{{\text{n}},{\text{t}} - 1}} } \\ \end{array} } \right]$$

4 $${\mathbf{U}} = \left[ {\begin{array}{*{20}c} {{\text{u}}_{1,2} } & \cdots & {{\text{u}}_{{{\text{n}},2}} } \\ \vdots & \ddots & \vdots \\ {{\text{u}}_{{1,{\text{t}}}} } & \cdots & {{\text{u}}_{{{\text{n}},{\text{t}}}} } \\ \end{array} } \right]$$

The output vector is defined as

5 $${\mathbf{y}} = \left[ {\begin{array}{*{20}c} {{\text{y}}_{1} } & \ldots & {{\text{y}}_{\text{t}} } \\ \end{array} } \right]^{\text{T}}$$

The *i*-*th* row **ai** for matrix **A** and vector **b** in (1) and (2) are obtained by solving the following equations where superscript T means vector transpose:

6 $${\mathbf{a}}_{\text{i}} = \left( {{\mathbf{U}}^{\text{T}} {\mathbf{U}}} \right)^{ - 1} {\mathbf{U}}^{\text{T}} {\text{x}}_{\text{i}}$$

7 $${\mathbf{b}} = \left( {{\mathbf{U}}^{\text{T}} {\mathbf{U}}} \right)^{ - 1} {\mathbf{U}}^{\text{T}} {\text{y}}$$

In order to adjust the Linear Predictive Adaptive Model, each day the procedure is repeated when new data is available. Every day, the first column of **X** and **U** matrix is removed and the new data added to the last column to generated a new set of coefficients.

## Results and discussion

### Characterization of onion waste

All analyses were made in triplicate. Carbon to nitrogen ratio is 15. According to Dioha [41] and Romano [16], this ratio is at the appropriate minimum for anaerobic bacteria because the increase in carbon content will increase carbon dioxide formation and high value of nitrogen will enhance the production of ammonia gas that could increase pH [41]. The onion waste had average moisture of 83.7 %. This result is lower than presented by Romano [17] (moisture content of 92.6 %) because in present study some pieces of peel of onion waste were used. However, moisture content was in accordance with Coventry [9], who found moisture content of 87 %. Therefore, lignin (16.1 % dry basis) and the sum of cellulose and hemicellulose (69.5 %) content in this study are higher than reported for Romano [17], 0.4 and 10 %, respectively.

### Coupled system performance

The First HPR1 after 63 days did not show evidence of methanogenic phase. Therefore, in the second experiment (HPR2), pretreatment was necessary with H_2_SO_4_. It reached the methanogenic phase on day 14 and the effluent was fed to the UASB reactor at constant OLR; this was achieved by varying feed volume.

**Fig. 2:**
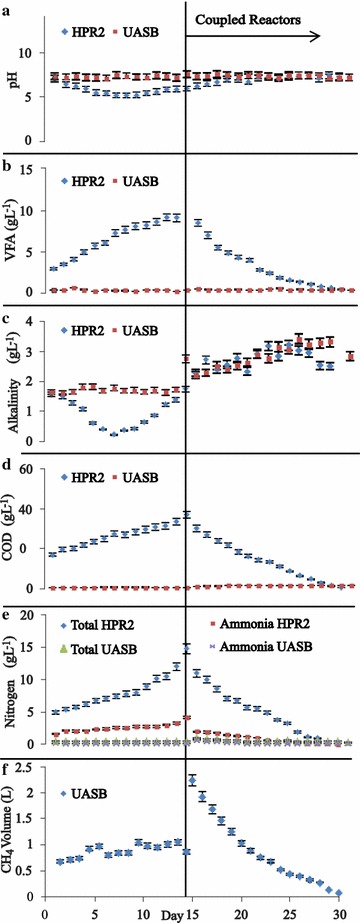
HPR2 + UASB reactors profiles: **a** pH; **b** VFA; **c** alkalinity; **d** COD; **e** nitrogen; **f** CH_4_ volume

Figure 2a shows the behavior of pH in the HPR2 + UASB reactors; hydrolysis pH falls quickly to an average value of 5.15 ± 0.29 at day 7. According to Kim [42], hydrolysis should be at pH 5.5 and Kapdan [43] mentioned a range between 5 and 6. At day 14, pH rose to 5.92 ± 0.9 where it reached the methanogenic phase. With the coupled system, it reached a pH near 7 within optimal limits for methane production due to consumption of VFA in the UASB. Furthermore, its effluent was recirculated. In the following days the pH of UASB reactor did not present significant change; it ranged from 7.1 ± 0.01 to 7.6 ± 0.04 which is ideal for anaerobic digestion process according to Mao [44].

Figure 2b shows VFA performance. The first days had an average value of 2.9 ± 0.9 gL^−1^; at day 14 the value was 11.2 ± 0.5 gL^−1^. After coupled, a rapid fall occurred and at the end of the experiment VFA concentration was 0.3 ± 0.1 gL^−1^ which is equivalent to a VFA reduction of 97 %. This behavior is in accordance with Lehtomäki [40], a reduction over 90 % indicating that this behavior was due to consumption by the bacterial consortium of the VFA entering the reactor.

Figure 2c shows alkalinity profile for HPR2 and UASB reactors. Hydrolysis reactor presented a decrease in the first 7 days with minimum value of 0.2 ± 0.2 gL^−1^. Chugh [45] mentioned that at low pH un-ionized species of VFAs are formed and consume the bicarbonate alkalinity. After coupled system, HPR2 reached a value of 1.7 ± 0.2 gL^−1^ at day 14. For UASB reactor before coupled system, alkalinity was maintained almost constant. After that an increase was presented, Chugh [45], which indicated the correct buffer capacity of the reactor.

The response for COD is shown in Fig. 2d. Hydrolysis reactor at day 14 reached a maximum of 31.2 ± 0.5 gL^−1^. At this point two reactors were coupled because a maximum COD and VFA were reached. The experiment ended when steady-state value was reached; this was the average of three consecutive measurements for COD when the deviations between the observed values were less than 5 %, according to Borja [46].

Figure 2e shows the profile for total and ammonia nitrogen; it shows total nitrogen peaking at 14 days at 14.7 ± 0.8 gL^−1^. On the same day maximum ammonia nitrogen concentration of 4.1 ± 0.2 gL^−1^ was reached. After connection, a fall in nitrogen profile was presented to finalize the experiment at 0.17 ± 0.07 gL^−1^ and 0.08 ± 0.07 gL^−1^ for total and ammonia nitrogen, respectively. This is equivalent to a removal efficiency of 98 % for both species. These results are similar to those reported by El-Kamah [47] where removal efficiencies of 72 ± 6 % and 99 ± 1.3 % for total and ammonium nitrogen, respectively, are shown. Yenigün [48] indicated that an ammonia nitrogen concentration over 1.7 gL^−1^ can inhibit anaerobic digestion for complex substrates without inoculum acclimation; however, the addition of small volumes in UASB reactor allows acclimation to the high concentration of ammonia nitrogen.

Methane volume profile for UASB reactor is presented in Fig. 2f. Before coupled reactor, it gets an average volume of 0.8 Ld^−1^; after coupled, it gets a maximum of 2.2 ± 0.5 L and decreased because the carbon source was exhausted. After, digestion hydrolysis reactors were emptied and measured for remaining VS and TS. The TS and VS reductions were determined and results were 55.7 ± 8 % and 83.5 ± 9.8 %, respectively. Romano [17] reported a reduction of VS of 62 ± 17 %, as shown in Table 1. This behavior indicates a better performance of the system with chemical pretreatment and coupling when COD and VFA reached a maximum value in the hydrolysis reactor.

**Table 1:** Anaerobic digestion of waste material in two-stage processes

Feedstock	System	Hydrolysis reactor				Methanogenic reactor	Two-stage		References
		OLR kg VS (m^3^d)^−1^	% TS	% VS	SRT (d)	HRT (d)	Methane yield m^3^ (kg VS_removed_)^−1^	VS removal %	
Vegetable waste	CSTR-AFBR	0.15	5.4 ± 0.6	4.9 ± 0.6	48	8	0.29 ± 0.01	91.0 ± 1.2	[49]
		0.1			43		0.33 ± 0.02	91.1 ± 2.4	
		0.17			44		0.33 ± 0.03	90.9 ± 2.8	
		0.23			45		0.31 ± 0.05	77.2 ± 1.8	
Fruit and vegetable waste	CSTR–CSTR	7.0	12.7 ± 0.9	11.0 ± 0.8	25	5	0.3	97.5	[50]
Tannery solid waste	CSTR–CSTR	1.05 ± 0.05	7.04	2.82	10	20	0.31	67	[51]
Activated sludge (84 %) + organic waste (16 %)	CSTR–CSTR	19.9	9.8	N.D.	28	8	0.24	71.3	[60]
Onion waste	SBR-AMBR	0.5	7.4	7.11	14	3	0.29	N.A.	[17]
		1				3	0.32	N.A.	
		2				3	0.31	N.A.	
Tomato	LBAR-UASB	6.7	10	7.6	31	N.A.	0.04	47	[61]
Cucumber		1.6	6.8	4.5			0.07	54	
Common reed		15.2	44.3	41			0.011	7.7	
Grass silage		14.5	41	39			0.011	31.6	
Blue mussel	LBAR-UASB	0.5	41.2	7.7	44	N.A.	0.33	N.A.	[62]
Red mussel		0.5	81.9	76.3	107		0.22	N.A.	
Vinegar residue + sludge pretreated with HCl	CSTR–CSTR	2.6	30.8	24.7	N.A	N.A.	0.192	N.A.	[52]
Sewage, pretreated with H_2_SO_4_	CSTR–CSTR	1.4	N.A.	N.A.	20	N.A.	0.45	59.6	[53]
Onion waste, pretreated with H_2_SO_4_	HPR2-UASB	2.7	8.65 ± 0.29	6.83 ± 0.23	30	12	0.43 ± 0.06	83.5 ± 9.8	This study

*OLR* Organic load rate, *SRT* Solid retention time, *HRT* Hydraulic retention time, *CSTR* Continuous stirred tank reactor, *AFBR* Anaerobic fluidized bed reactor, *SBR* Solid bed reactor, *AMBR* Anaerobic membrane bed reactor, *LBAR* Leach bed anaerobic reactor, *N.A.* Not available

Average cumulative methane yield for UASB reactor after coupled system was 0.43 ± 0.06 m^3^ (kg VS_rem_)^−1^ and 76 % of the methane content. As is presented in Table 1, this result is slightly higher than reported by Romano [17], 0.3 m^3^ (kg VS_rem_)^−1^. The methane yield reported in the present study is comparable with other authors such as Zuo [49] vegetable wastes 0.31 ± 0.2 (kg VS_rem_)^−1^; Ganesh [50] vegetable and fruit waste 0.301 m^3^ (kg VS_rem_)^−1^ and Arumugam [51] 0.31 m^3^ (kg VS_rem_)^−1^.

With regard to studies where pretreatment of the residue was performed, the yield of methane is greater than reported by Wang [52] where for anaerobic digestion in two phases with pretreatment with HCl yield was 0.192 m^3^ (kg VS_rem_)^−1^. However, yield reported by Takashima [53] was 0.45 m^3^ (kg VS_rem_)^−1^. They indicated that the behavior is attributed to the release of cellulose and hemicellulose due to acid pretreatment.

The degradation of 90 % VS was achieved in 30 days; for the HPR2 + UASB system it was higher than reported by Romano [17], which reported 14 days. The main reason for this difference is that Romano defined a maximum process time of 14 days. In other studies, time reported is in accordance with this study. Zuo [49] reported between 44 and 48 days for more than 90 % VS removal; Ganesh [50] 25 days for 97.5 % VS removal; Arumugam [51] 10 days for 67 % removal.

### Linear predictive adaptive model (LPAM)

**Fig. 3:**
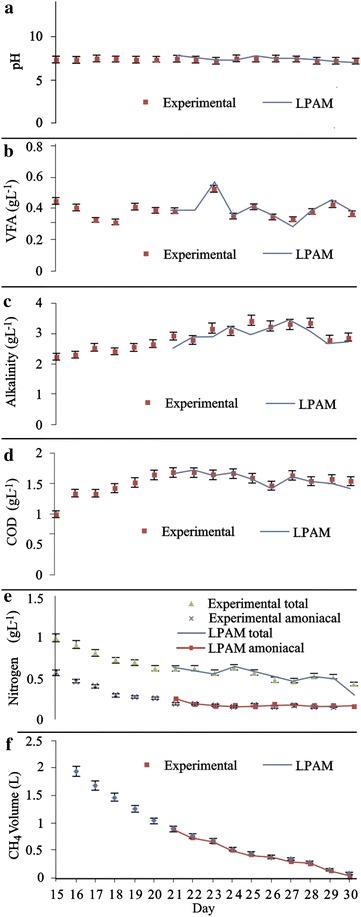
Experimental (*dotted mark*) and predicted (*solid line*) values of various parameters used as state in the model, including: **a** pH; **b** VFA; **c** alkalinity; **d** COD; **e** nitrogen; **f** CH_4_ volume

The model was developed once the two reactors were coupled at day 14 in accordance with COD and VFA criterions defined before. As the model needs a reference set of variables, data from day 14 to 19 were used to build **X** and **U** matrix. The prediction of methane generation and other parameters started on day 20 of the experiment. The experimental value and its prediction of daily methane generation and all state parameters are shown by the dotted mark and solid lines in Fig. 3.

A good correlation between the daily data and the results of the model prediction was observed as shown in Fig. 3. This not only reflects the strength of LPAM, which although simple is quite useful, but also establishes a biological process which remained constant OLR as a union factor between the reactors. As shown in Fig. 3a, pH has excellent fit; this is due to the low variability in UASB reactor. On the other hand, the model estimation on VFA and Alkalinity occasionally showed significant increase and decrease which is similar behavior for experimental and model data, Fig. 3c, d. As mentioned by Ahn [54], an addition of VFA reduces the alkalinity, but methanogens consume VFA and generate alkalinity.

LPAM applied to COD; total and ammonia nitrogen profiles had a good correlation as shown in Fig. 3e–g, respectively. As Sendjaja [32] mentioned, even though profiles show good prediction of various parameters, a quantitative measure is necessary. For this reason the accuracy of prediction values was evaluated by comparing the area bound by curves from predicted and experimental values and X-axis. The error value is defined as the ratio between the difference in total area and the area bound by curve from experimental value; results are summarized in Table 2.

**Table 2:** Area under the curve for experiment and predicted value vs time

Parameter	V CH_4_ (L)	COD (gL^−1^)	PH	VFA acetic acid (gL^−1^)	Alkalinity (CaCO_3_ g L^−1^)	Total nitrogen (gL^−1^)	Ammonia nitrogen (gL^−1^)
Experiment	17.02	22.07	146.88	7.18	47.28	11.79	4.47
Prediction	16.92	21.91	147.27	7.28	46.41	11.75	4.50
Error (%)	0.61	0.74	0.26	1.48	1.84	0.27	0.69

As shown in Table 2, maximum error was for alkalinity model with 1.84 %, followed by VFA (1.48 %), COD (0.74 %), ammonia nitrogen (0.69 %), methane volume (0.61 %), total nitrogen (0.27 %) and finally pH (0.26 %). These results are in agreement with the results presented by Sendjaja [32] where the error for UASB reactor for VFA was 7.09 %; COD was 2.33 %; pH was 0.59 % and methane volume was 7.26 %.

Table 3 shows the summary of various studies using mathematical models applied to anaerobic digestion. A ANFIS model for prediction of anaerobic digestion effluent quality was applied to a UASB. The authors indicated that enlarging of the database and/or frequency of monitoring will serve to reduce the error level and improve the predicting capability of the model; however, it was used for the entire experiment (85 days) [31]. Cakmakci [55] used ANFIS models to predict effluent versus concentration and methane yield in the anaerobic digester of primary sedimentation sludge. Due to highly nonlinear structure of the ANFIS model, a complex system such as anaerobic digestion could be easily modeled was shown; however, a large quantity of data was necessary for training zone (135 days) and testing zone (35 days). For model ANFIS, one learning stage is required and another for verification model, whereby the number of required data is quite high. This is in agreement with Sendjaja [32] where it is mentioned that one of the weaknesses of the models based on historical data is the need to obtain a lot of data, as well as maintaining stability in the process.

**Table 3:** Comparison between various model applications for anaerobic digestion

Reactor	Model	System	Number of data	Constant supposed	Independent variables	Reference
UASB	ANFIS	Continuous	85	No	pH, COD, VFA	[31]
CSTR	ANFIS	Continuous	165	No	pH, influent VS concentration, temperature	[55]
CSTR	ADM1	Continuous	20	Yes	AGV, yield	[56]
CSTR	AMD1	Continuous	140	Yes	pH, inorganic nitrogen, gas flow rate, COD	[57]
CSTR - UASB	AMD1	Continuous	120	Yes	COD, pH, VFA	[58]
LBAR	AMD1	Batch	81	Yes	VFA, biogas volume, methane concentration, pH	[59]
CSTR - CSTR	‘‘Adaptive’’ discrete state space model	Continuous	85	No	VFA, total COD, soluble COD, total solids, pH, methane volume	[32]
HPR2-UASB	LPAM	Semi-continuous	30	No	VFA, COD, total nitrogen, ammonium nitrogen, alkalinity, methane volume	This study

*CSTR* Continuous stirred tank reactor, *LBAR* Leach bed anaerobic reactor

Zhao [56] developed AMD1 model for anaerobic digestion of lignocellulose-rich aquatic plants. Experiments were carried out to identify the parameters and calibrate and validate this model. The values were calculated and estimated for 7 of the 15 stoichiometric parameters and 9 of the 17 kinetic parameters, respectively. The model validation required 20 days.

Mairet [57] proposed a modified version of AMD1 (based on Contois kinetics for hydrolysis steps) to represent microalgae anaerobic digestion. After 140 days of experimentation, a good representation of the data was obtained, including pH inhibition. Chen [58] implemented a mathematical model to simulate two-phase anaerobic digestion process treating traditional Chinese medicine wastewater with AMD1. It was necessary to estimate stoichiometric coefficients, equilibrium coefficients, and kinetic parameters using an explicit Runge–Kutta pair of Bogacki and Shampine. However, it failed to simulate the CSTR performance after 120 days of experiment. For AMD1 models it is necessary to assume some constants which can vary according to the process conditions; others have to be calculated or estimated from experimental data. Similarly, a high number of data is needed in order to adjust some parameters not found in the literature. Sendjaja [32] indicates that these models require several parameters which must be assumed or determined from experimental data. Also, these constants are assumed to be the same throughout the experiment, which is not entirely true mainly due to the variation of internal or external conditions.

## Conclusions

Anaerobic digestion of onion waste in two stages can be a good alternative for reducing the residues and generating methane. Pretreatment was necessary to accelerate the digestion, lead to faster production of biogas and obtain a major methane concentration. The system reached a yield of methane of 0.43 (kg VS_rem_)^−1^. VFA, COD, total and ammonia nitrogen reductions were 93.6, 98, 98.7 and 95 %, respectively.

It is necessary to keep OLR constant as coupling parameter between HPR2 and UASB, avoid poisoning the UASB reactor, allow acclimation of UASB reactor to HPR2 effluent, and not require the addition of external buffer for UASB reactor.

A predictive adaptive linear model for anaerobic digestion was developed for the coupled HPR2 + UASB reactors. The model was able to predict the performance of the anaerobic process, including methane volume, COD, pH, VFA, alkalinity, total and ammonia nitrogen. The behavior predicted by the model can be a tool to establish control procedures in order to ensure suitability for the production of methane conditions.

One of the strengths of the proposed LAPM is the application to a system without the need for a very broad range of experimental data besides daily adaptation to internal and external conditions that the reactor is experiencing. The use of constants found in the literature is not required so that the error that is committed is minimized; further experiments to adjust, for example AMD1 model, parameters are reduced.

## Abbreviations

OLR: organic load rate
VFA: volatile fatty acids
UASB: upflow anaerobic stirred bed reactor
VS: volatile solids
AMBR: anaerobic membrane bed reactor
LPAM: linear predictive adaptive model
MC: moisture content
TS: total solids
TCD: thermal conductivity detector
HPR1: hydrolysis reactor single-phase
HPR2: hydrolysis reactor with acid pretreatment
COD: chemical organic demand
SW: synthetic wastewater
SRT: solid retention time
HRT: hydraulic retention time
AFBR: anaerobic fluidized bed reactor
SBR: solid bed reactor
LBAR: leach bed anaerobic reactor
NA: not available
ANFIS: adaptive neuro fuzzy inference system
AMD1: anaerobic digestion model No. 1

## References

[1] FAOSTAT. Food and Agricultural commodities production. http://faostat3.fao.org/home/E. Accessed 15 Jul 2016.

[2] Servicio de Información Agroalimentaria y Pesquera. Anuario Estadístico de la Producción Agrícola. http://infosiap.siap.gob.mx/aagricola_siap/icultivo/index.jsp. Accessed 15 Mar 2016.

[3] Mahdizadeh Khasraghi M, Gholami Sefidkouhi MA, Valipour M (2015). Simulation of open-and closed-end border irrigation systems using SIRMOD. Archives Agron Soil Sci.

[4] Valipour M (2012). Sprinkle and trickle irrigation system design using tapered pipes for pressure loss adjusting. J Agric Sci.

[5] Yannopoulos SI, Lyberatos G, Theodossiou N, Li W, Valipour M, Tamburrino A, Angelakis AN (2015). Evolution of water lifting devices (pumps) over the centuries worldwide. Water.

[6] Benítez V, Mollá E, Martín-Cabrejas MA, Aguilera Y, López-Andréu FJ, Cools K, Terry LA, Esteban RM (2011). Characterization of industrial onion wastes (Allium cepa L.): dietary fibre and bioactive compounds. Plant Food Hum Nutr.

[7] Prakash D, Singh BN, Upadhyay G (2007). Antioxidant and free radical scavenging activities of phenols from onion (Allium cepa). Food Chem.

[8] Martínez RM, Miglierina AM, Luna M (2008). Konijnenburg Av, Pellejero G: evalución del compostaje de los residuos del procesamiento de cebolla. Pilquen Secc Agron.

[9] Coventry E, Noble R, Mead A, Whipps JM (2002). Control of Allium white rot (Sclerotium cepivorum) with composted onion waste. Soil Biol Biochem.

[10] Rose P, Whiteman M, Moore PK, Zhu YZ (2005). Bioactive S-alk(en)yl cysteine sulfoxide metabolites in the genus Allium: the chemistry of potential therapeutic agents. Nat Prod Rep.

[11] Clarkson JP, Scruby A, Mead A, Wright C, Smith B, Whipps JM (2007). Integrated control of Allium white rot with Trichoderma viride, tebuconazole and composted onion waste. Plant Pathol.

[12] Horiuchi JI, Kanno T, Kobayashi M (1999). New Vinegar Production from Onions. J Biosci Bioeng.

[13] Horiuchi JI, Tada K, Kobayashi M, Kanno T, Ebie K (2004). Biological approach for effective utilization of worthless onions - vinegar production and composting. Resour Conserv Recycl.

[14] Horiuchi JI, Kanno T, Kobayashi M (2000). Effective onion vinegar production by a two-step fermentation system. J Biosci Bioeng.

[15] Mallek SB, Prather TS, Stapleton JJ (2007). Interaction effects of Allium spp. residues, concentrations and soil temperature on seed germination of four weedy plant species. Appl Soil Ecol.

[16] Romano RT, Zhang R (2008). Co-digestion of onion juice and wastewater sludge using an anaerobic mixed biofilm reactor. Bioresour Technol.

[17] Romano RT, Zhang R (2011). Anaerobic digestion of onion residuals using a mesophilic anaerobic phased solids digester. Biomass Bioenerg.

[18] Kothari R, Tyagi V, Pathak A (2012). Waste-to-energy: a way from renewable energy sources to sustainable development. Renew Sustain Energy Rev.

[19] Gunaseelan VN (2004). Biochemical methane potential of fruits and vegetable solid waste feedstocks. Biomass Bioener.

[20] Menardo S, Balsari P (2012). An analysis of the energy potential of anaerobic digestion of agricultural by-products and organic waste. Bioener Resour.

[21] Nasir IM, Ghazi TIM, Omar R (2012). Production of biogas from solid organic wastes through anaerobic digestion: a review. Appl Microbiol Biotechnol.

[22] Wang Y, Zhang Y, Wang J, Meng L (2009). Effects of volatile fatty acid concentrations on methane yield and methanogenic bacteria. Biomass Bionener.

[23] Kim W, Shin S, Cho K, Lee C, Hwang S (2012). Performance of methanogenic reactors in temperature phased two-stage anaerobic digestion of swine wastewater. J Biosci Bioeng.

[24] Sridevi VD, Rema T, Srinivasan SV (2015). Studies on biogas production from vegetable market wastes in a two-phase anaerobic reactor. Clean Technol Environ Polic.

[25] Cooney M, Maynard N, Cannizzaro C, Benemann J (2007). Two-phase anaerobic digestion for production of hydrogen–methane mixtures. Bioresour Technol.

[26] Yu H, Samani Z, Hanson A, Smith G (2002). Energy recovery from grass using two-phase anaerobic digestion. Waste Manag.

[27] Lenihan P, Orozco A, O’Neill E, Ahmad MNM, Rooney DW, Walker GM (2010). Dilute acid hydrolysis of lignocellulosic biomass. Chem Eng J.

[28] Lima CSS, Conceição MM, Silva FLH, Lima EE, Conrado LS, Leão DAS (2013). Characterization of acid hydrolysis of sisal. Appl Energy.

[29] Duarte JG, Silva LLS, Freire DMG (2015). M.C. Cammarota, Gutarra MLE: enzymatic hydrolysis and anaerobic biological treatment of fish industry effluent: Evaluation of the mesophilic and thermophilic conditions. Renew Energy.

[30] Lubberding H, Gijsen H, Heck M (1988). G V: anaerobic digestion of onion waste by means of rumen microorganisms. Biol Wastes.

[31] Erdirencelebi D, Yalpir S (2011). Adaptive network fuzzy inference system modeling for the input selection and prediction of anaerobic digestion effluent quality. Appl Math Model.

[32] Sendjaja A, Tan Y, Pathak S, Zhou Y, Abdul M, Liu J, Ng W (2015). Regression based state space adaptive model of two-phase anaerobic reactor. Chemosphere.

[33] Standard methods for the examination of water and wastewater. Washington: American Public Health Association; 1998. Accessed 29 Jan 2015.

[34] España-Gamboa E, Mijangos-Cortes J, Hernández-Zárate G, Domínguez-Maldonado J, Alzate-Gaviria L (2012). Methane production by treating vinasses from hydrous ethanol using a modified UASB reactor. Biotechnol Biofuels.

[35] Purser BJJ, Thai S-M, Fritz T, Esteves SR, Dinsdale RM, Guwy AJ (2014). An improved titration model reducing over estimation of total volatile fatty acids in anaerobic digestion of energy crop, animal slurry and food waste. Water Res.

[36] Blanco-Cocom L, Guerrero-Alvarez A, Domınguez-Maldonado J, Avila-Vales E, Alzate-Gaviria L (2013). Mathematical model for a continuous hydrogen production system: stirred fermenter connected to a biocatalyzed electrolysis cell. Biomass Bioener.

[37] Alzate-Gaviria LM, Sebastian PJ, Pérez-Hernándezc A, Eapen D (2007). Comparison of two anaerobic systems for hydrogen production from the organic fraction of municipal solidwaste and syntheticwastewater. Int J Hydrog Energy.

[38] Chen X, Romano RT, Zhang R (2010). Anaerobic digestion of food wastes for biogas production. Int J Agric Biol Eng.

[39] Rajeshwari KV, Balakrishnan M, Kansal A, Lata K, Kishore VVN (2000). State-of-the-art of anaerobic digestion technology for industrial wastewater treatment. Renew Sustainable Energy Rev.

[40] Lehtomaki A, Huttunen S, Lehtinen TM, Rintala JA (2008). Anaerobic digestion of grass silage in batch leach bed processes for methane production. Bioresour Technol.

[41] Dioha IJ, Ikeme CH, Nafi T, Soba NI (2013). Effect of carbon to nitrogen ratio on biogas production. Int Res J Nat Sci.

[42] Kim J, Par C, Kim T, Lee M, Kim S, Kim S, Lee J (2002). Effects of various pretreatments for enhanced anaerobic digestion with waste activated sludge. J Biosci Bioeng.

[43] Kapdan I, Kargi F (2006). Bio-hydrogen production from waste materials. Enzym Microb Technol.

[44] Mao C, Feng Y, Wang X, Ren G (2015). Review on research achievements of biogas from anaerobic digestion. Renew Sustain Energy Rev.

[45] Chugh S, Chynoweth DP, Clarke W, Pullammanappallil P, Rudolph V (1999). Degradation of unsorted municipal solid waste by a leach-bed process. Bioresour Technol.

[46] Borja R, Sánchez E, Rincon B, Raposo F, Martin MA, Martın A (2005). Study and optimisation of the anaerobic acidogenic fermentation of two-phase olive pomace. Process Biochem.

[47] El-Kamah H, Mahmoud M, Tawfik A (2011). Performance of down-flow hanging sponge (DHS) reactor coupled with up-flow anaerobic sludge blanket (UASB) reactor for treatment of onion dehydration wastewater. Bioresour Technol.

[48] Yenigün O, Demirel B (2013). Ammonia inhibition in anaerobic digestion: a review. Process Biochem.

[49] Zhuang Zuo SW (2014). Wanqin Zhang, Renjie Dong: performance of two-stage vegetable waste anaerobic digestion depending on varying recirculation rates. Bioresour Technol.

[50] Ganesh R, Torrijos M, Sousbie P, Lugardon A, Steyer JP, Delgenes JP (2014). Single-phase and two-phase anaerobic digestion of fruit and vegetable waste: comparison of start-up, reactor stability and process performance. Waste Manag.

[51] Arumugam T, Parthiban L, Rangasamy P (2015). Two-phase anaerobic digestion model of a tannery solidwaste: experimental investigation and modeling with ANFIS. Arab J Sci Eng.

[52] Wang Z, Shao S, Zhang C, Lu D, Ma H, Ren X (2015). Pretreatment of vinegar residue and anaerobic sludge for enhanced hydrogen and methane production in the two-stage anaerobic system. Int J Hydrog Energy.

[53] Takashima M, Tanaka Y (2010). Application of acidic thermal treatment for one- and two-stage anaerobic digestion of sewage sludge. Water Sci Technol.

[54] Ahn HK, Smith MC, Kondrad SL, White JW (2010). Evaluation of biogas production potential by dry anaerobic digestion of switchgrass-animal manure mixtures. Appl Biochem Biotechnol.

[55] Cakmakci M (2007). Adaptive neuro-fuzzy modelling of anaerobic digestion of primary sedimentation sludge. Bioprocess Biosyst Eng.

[56] Zhao B-H, Yue Z-B, Ni B-J, Mu Y, Yu H-Q, Harada H (2009). Modeling anaerobic digestion of aquatic plants by rumen cultures: cattail as an example. Water Res.

[57] Mairet F, Bernard O, Ras M, Lardon L, Steyer J-P (2011). Modeling anaerobic digestion of microalgae using ADM1. Bioresour Technol..

[58] Chen Z, Hu D, Zhang Z, Ren N, Zhu H (2009). Modeling of two-phase anaerobic process treating traditional chinese medicine wastewater with the iwa anaerobic digestion model no. 1. Bioresour Technol.

[59] Lai TE, Koppar AK, Pullammanappallil PC, Clarke WP (2009). Mathematical modeling of batch, single stage. Leach bed anaerobic digestion of organic fraction of municipal solid waste.

[60] Schievano A, Tenca A, Scaglia B, Merlino G, Rizzi A, Daffonchio D, Oberti R, Adani F (2012). Two-stage vs single-stage thermophilic anaerobic digestion: comparison of energy production and biodegradation efficiencies. Environ Sci Technol.

[61] Jagadabhi PS, Kaparaju P, Rintala J (2011). Two-stage anaerobic digestion of tomato, cucumber, common reed and grass silage in leach-bed reactors and upflow anaerobic sludge blanket reactors. Bioresour Technol.

[62] Nkemka VN, Murto M (2013). Two-stage anaerobic dry digestion of blue mussel and reed. Renew Energy.

